# Prevalence and Impact of Urinary Incontinence at 5–10 Years After a Singleton Birth

**DOI:** 10.3390/jcm15010252

**Published:** 2025-12-29

**Authors:** Lola Serrano-Raya, Ana Esplugues, Inmaculada Ferreros Villar, Nerea Vallés-Murcia, Paula Muñoz Esteban, María Sol Torres López, Elisa Turrión Martínez, Patxi Errandonea García, Francisco Jose Nohales Alfonso, Alba González-Timoneda

**Affiliations:** 1 Midwifery Training Unit, Valencian Community, Valencian School of Health Studies, Regional Ministry of Health, Calle Juan de Garay 21, 46017 Valencia, Spain; serrano_marray@gva.es; 2 Department of Nursing, Faculty of Health Sciences, Universidad Cardenal Herrera-CEU, CEU Universities, Calle Santiago Ramon y Cajal 15, Alfara del Patriarca, 46115 Valencia, Spain; 3 Department of Nursing, Faculty of Nursing and Podiatry, University of Valencia, Avenida Menéndez y Pelayo 19, 46010 Valencia, Spain; gonzalez_albtim@gva.es; 4 Epidemiology and Environmental Health Joint Research Unit, FISABIO−Universitat Jaume I−Universitat de València, Blasco Ibáñez Campus, Avda Cataluña 21, 46020 Valencia, Spain; 5 Spanish Consortium for Research on Epidemiology and Public Health (CIBERESP), Av. Monforte de Lemos, 3-5. Pabellón 11. Planta 0, 28029 Madrid, Spain; 6 Health Information Analysis Service, Regional Ministry of Health, Calle Micer Mascó 31-33, 46010 Valencia, Spain; ferreros_inm@gva.es; 7 Neonatal Research Group, La Fe Health Research Institute, La Fe University and Polytechnic Hospital, Avenida Fernando Abril Martorell 106, 46026 Valencia, Spain; valles_ner@gva.es; 8 Department of Obstetrics and Gynecology, La Fe University and Polytechnic Hospital, Avenida Fernando Abril Martorell 106, 46026 Valencia, Spain; pmeaula98@gmail.com (P.M.E.); torres_mariasollop@gva.es (M.S.T.L.); turrion_mel@gva.es (E.T.M.); perrandonea@santpau.cat (P.E.G.); 9 Gynecology (Pelvic Floor) Section, La Fe University and Polytechnic Hospital, Avenida Fernando Abril Martorell 106, 46026 Valencia, Spain; fnohalesa@gmail.com; 10 Research Group on Maternal Cardiovascular Health, Preeclampsia and Preterm Birth, La Fe Health Research Institute, La Fe University and Polytechnic Hospital, Avenida Fernando Abril Martorell 106, 46026 Valencia, Spain

**Keywords:** urinary incontinence, risk factors, vaginal delivery, primiparity, quality of life

## Abstract

**Background/Objectives:** To analyze the prevalence and risk factors associated with the onset of urinary incontinence (UI) in primiparous non-menopausal women with no personal history of pregestational UI, as well as its impact on quality of life. **Methods:** An ambispective observational cohort study was conducted among primiparous women between 5 and 10 years after childbirth. Sociodemographic and health characteristics were analyzed, along with the presence of UI using the *International Consultation on Incontinence Questionnaire–Urinary Incontinence Short Form* (ICIQ-UI-SF). **Results:** Of the 425 women analyzed, 228 (53.6%) presented UI. After adjusting for confounding factors, women who delivered by cesarean section showed a lower risk of developing UI (aOR = 0.52; 95% CI: 0.32–0.85; *p* = 0.009). Conversely, a family history of UI (mother or sister) (aOR = 2.03; 95% CI: 1.25–3.32; *p* = 0.004) and the presence of medical history (chronic diseases/comorbidities) were associated with a higher risk of UI (aOR = 1.58; 95% CI: 1.02–2.45; *p* = 0.040). Regarding quality of life, 25.88% of participants responded affirmatively to the specific interview question on UI, whereas 58.65% presented some degree of UI when assessed with the ICIQ-UI-SF. This discrepancy likely corresponds to mild-to-moderate cases. **Conclusions:** In primiparous, non-menopausal women without prior incontinence, the occurrence of UI in subsequent years following childbirth is associated with vaginal delivery, family history of UI, and the presence of chronic diseases or comorbidities. Evidence-based strategies for detection and prevention should be further developed and implemented.

## 1. Introduction

Urinary incontinence (UI)—defined by the International Continence Society (ICS) as any involuntary loss of urine—is one of the most prevalent women’s health problems, with a significant social and economic burden [1,2,3]. UI is one of the most frequent pelvic floor disorders (PFDs) in women and constitutes a significant public health problem. Although it is not a severe pathology, UI has a marked negative impact on women’s health-related quality of life (HRQoL), affecting their physical, psychological, social, and sexual well-being, with repercussions extending beyond the urological domain [3,4,5,6,7]. Its impact is comparable to that of other chronic diseases, as it interferes with daily activities, rest, emotional state, and interpersonal relationships [3,4,7,8,9]. UI usually follows a chronic course, and its prevalence increases with age and hormonal decline, particularly during the climacteric period [10]. Since 1998, the World Health Organization (WHO) has recognized it as a distinct entity in the International Classification of Diseases (ICD), which has promoted standardization and epidemiological surveillance [11]. In Europe, prevalence rates ranging from 14.1% to 68.8% in women and from 8.3% to 23.7% in those under 50 years of age have been reported [7,12].

The “Lifespan Model” proposed by DeLancey et al. (2008) [13] serves as a conceptual framework integrating the causal factors of pelvic floor disorders (PFDs). It offers a comprehensive approach to understanding the multifactorial etiology of pelvic floor dysfunctions. The model posits that these disorders result from the cumulative interaction of predisposing factors (genetic and developmental), initiating factors (particularly obstetric events such as vaginal delivery), and intervening factors (aging, menopause, obesity, or lifestyle habits), which progressively diminish the pelvic floor’s functional reserve throughout the lifespan until the symptomatic threshold is surpassed.

DeLancey emphasizes the importance of integrating individual and life-course factors in the prevention and management of these dysfunctions. The primiparous model is considered the most appropriate for research, as it allows for the study of the impact of the first delivery on an intact pelvic floor and the isolation of the initial mechanisms of injury and recovery without the confounding influence of previous deliveries or aging [13].

According to the ICS, UI is classified into three main types: stress, urge, and mixed urinary incontinence. Stress urinary incontinence (SUI) is defined as the involuntary leakage of urine associated with an increase in intra-abdominal pressure during physical exertion, coughing, or sneezing. SUI is the most common subtype, with well-established risk factors such as age, body mass index (BMI), parity, vaginal birth, obesity, menopause, smoking and chronic constipation [2,5,14,15,16,17,18,19]. After delivery many cases improve, but a significant proportion persist or reappear, and vaginal delivery remains a major risk factor for postpartum SUI [20,21,22,23,24]. At five years postpartum, population-based cohorts such as EPINCONT and the Viktrup study report SUI prevalence rates of approximately 20–25% following the first vaginal delivery [10,25]. Urge urinary incontinence (UUI), characterized by involuntary urine loss preceded or accompanied by a sudden and compelling desire to void, is usually associated with detrusor overactivity and factors such as diabetes mellitus, hypertension, obesity, high caffeine intake, diuretic use, and depression [2,14]. Mixed urinary incontinence (MUI) generally results from a combination of the same factors that cause SUI and UUI. Additional contributing factors include estrogen deficiency typical of postmenopausal and older women, histomorphological abnormalities, and structural changes associated with obstetric or pelvic surgical history [2,13,14,26].

The natural history of SUI is influenced by multiple factors: previous UI (before or during pregnancy), elevated pregestational BMI (≥24 kg/m^2^), excessive gestational weight gain, prolonged second stage of labor, family history of UI [22,27,28,29,30,31] and the first delivery appears to be the most determinant event [28]. In the current demographic context, with a mean maternal age at first birth of 32.59 years in Spain [32], age-related vulnerability may further increase the burden of UI. The most reliable estimates are obtained using standardized and validated instruments that allow classification by type and severity [33]. In Spain, according to the review by Salinas et al. (2010), UI affects approximately 24% of women, to increasing in frequency 30–40% in middle-aged women and up to 50% in older women [34].

Scientific evidence indicates that the severity and type of UI determine the degree of impact on quality of life. Women with MUI generally experience more severe symptoms and greater impairment than those with SUI or UUI [5]. In the national context, UI has been shown to limit daily activities, interpersonal relationships, and emotional well-being, particularly among older women or those with persistent symptoms [4].

Evidence supports the use of conservative interventions as the first-line treatment, including lifestyle and behavioral modifications such as weight control, avoidance of excessive fluid intake and consumption of caffeine or tea, and pelvic floor physiotherapy, due to their safety, accessibility, and favorable cost-effectiveness ratio [35].

Despite the abundance of studies addressing UI during pregnancy and the short-term postpartum period, evidence on its medium-term course remains limited and heterogeneous, particularly between 5 and 10 years after a first singleton delivery. This period is clinically relevant due to its impact on health-related quality of life (HRQoL).

The main objective of the present study was to estimate the prevalence of UI within this time interval. Secondary objectives included quantifying its impact on HRQoL and analyzing maternal, gestational, and delivery-related factors associated with its occurrence. By focusing on primiparous women and employing validated measurement tools, this study aims to provide useful estimates to guide prevention, screening, and conservative intervention strategies in healthcare practice.

## 2. Materials and Methods

### 2.1. Study Design and Participants

An ambispective observational cohort study was conducted to analyze the prevalence and risk factors associated with the onset of urinary incontinence (UI) between 5 and 10 years after the first delivery, as well as its impact on quality of life in these women.

The study population consisted of primiparous women who had given birth vaginally or by cesarean section at the La Fe University and Polytechnic Hospital (Valencia, Spain) between 2012 and 2016, and who had not experienced subsequent pregnancies. Women with a history of UI prior to pregnancy or who were postmenopausal at the time of selection were excluded. Additional exclusion criteria included multiple pregnancy, perinatal death, and minors.

**Figure 1:**
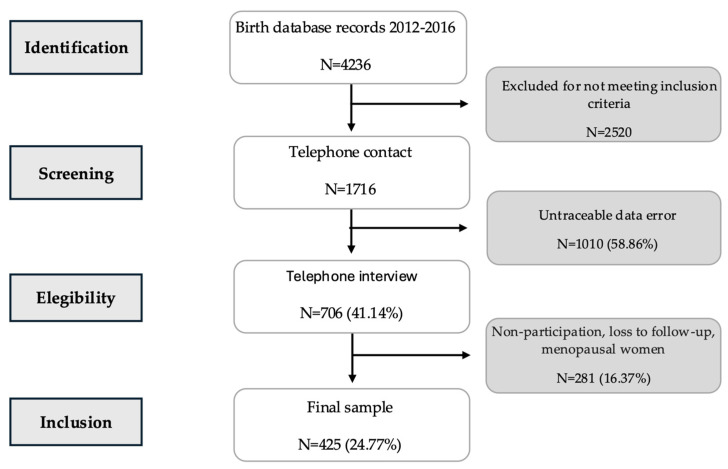
Flowchart depicting the selection process of women who gave birth to a single child between 2012 and 2016, as recorded in the La Fe University and Polytechnic Hospital database.

The population selection process is shown in Figure 1 using a flowchart based on the inclusion and exclusion criteria described above.

### 2.2. Data Collection

This sub-analysis was based on a population database derived from a larger study on pelvic floor dysfunctions five years after the first delivery, conducted at La Fe University and Polytechnic Hospital in Valencia, Spain [36].

Data were collected in two phases. First, a retrospective review of electronic medical records was performed to identify eligible participants and extract baseline information, including contact details, age, and confirmation of the absence of additional pregnancies recorded in the medical history up to the time of data collection.

In the second phase, eligible women were contacted by telephone and invited to participate after verifying compliance with the inclusion criteria and obtaining informed consent. Data collection was conducted through structured telephone interviews administered by nurse specialists in obstetrics and gynecology (midwives).

During the interviews, sociodemographic, health-related, obstetric, and pelvic floor variables were obtained using an ad hoc questionnaire. Outcome variables were assessed using the validated Spanish version of the International Consultation on Incontinence Questionnaire–Urinary Incontinence Short Form (ICIQ-UI-SF) a specific instrument designed to evaluate urinary incontinence symptoms and their impact on quality of life [37]. All questionnaires were administered during the telephone interviews, and no questionnaires were distributed separately.

Data collection was carried out between January 2022 and February 2023.

### 2.3. Variables and Operational Definitions

Urinary incontinence was defined according to the recommendations of the International Continence Society and the International Urogynecological Association (IUGA) as the “complaint of any involuntary loss of urine” [2].

Maternal BMI—pre-pregnancy, at delivery, and current—was categorized according to WHO classification as: underweight (<18.5 kg/m^2^), normal weight (18.5–24.9), overweight (25–29.9), and obese (≥30) [38].

In terms of educational level, a woman was considered to have a “high level of education” if she had completed vocational training or undergraduate, graduate, or postgraduate university studies, while those participants with elementary education or no education were classified as having a “low level of education.”

Physical activities were categorized as “low-impact” if at least one foot remained in contact with the ground (e.g., walking, swimming, cycling, yoga, or Pilates), and “high-impact” if both feet were in the air simultaneously at times (e.g., running or jumping) [39].

Women were classified as “without occupational physical load” if their work was sedentary or involved low physical demand, and as “with occupational physical load” if their job required moderate or high physical exertion [40].

Family history of UI was collected as a dichotomous variable. It was defined as the self-reported presence of urinary incontinence in first and second-degree female relatives, specifically the participant’s mother and/or sisters. No other family relationships were considered for this variable.

Medical history was collected as a categorical variable. In accordance with previously published literature, chronic medical conditions were defined as the presence of diabetes mellitus, hypertension, obesity, current use of diuretics, psychiatric disorders requiring pharmacological treatment, chronic obstructive pulmonary disease, or asthma [2,14]. Only conditions present at the time of data collection were considered for analysis.

The evaluation of UI was performed using the validated Spanish version of ICIQ-UI-SF, culturally adapted by Espuña et al. (2004) [41]. This questionnaire, widely used in both clinical practice and research, is designed for the detection and quantification of UI in healthcare settings. It consists of three main items assessing the frequency of urine leakage, the amount of urine lost, and the perceived impact on daily life. Additionally, it includes a final item with eight descriptive options aimed at identifying the type of UI, which are not included in the total score and are used solely for clinical orientation.

The total ICIQ-UI-SF score is obtained by summing the first three items, with a range of 0 to 21 points. In this study, all women with a total score of 0 were considered continent, and those with a score higher than 0 were considered to have some degree of urinary incontinence.

As validated cut-off points for symptom severity are not routinely established for ICIQ questionnaires, severity categories were defined in accordance with previous empirical research. Following the classification proposed by Klovning et al., ICIQ-UI-SF scores were categorized as mild (1–5), moderate (6–12), severe (13–18), and very severe (19–21) [42].

In this way, the ICIQ-UI-SF allowed not only to identify the presence of UI, but also to stratify the intensity of symptoms and assess their impact on quality of life.

### 2.4. Statistical Analysis

Statistical analyses were performed using Stata Statistical Software, version 16 (StataCorp LP, College Station, TX, USA). A general description of the sample was conducted using descriptive statistics for all variables, and prevalence rates were calculated with their corresponding 95% confidence intervals (95% CI).

Categorical variables were compared using the Chi-square (χ^2^) test, while continuous variables were compared using Student’s *t*-test.

Subsequently, bivariate and multivariate logistic regression analyses was performed to identify independent risk factors associated with the presence of urinary incontinence (UI). Variables included in the model were: age at delivery, current age, current BMI, type of delivery, and medical and family history.

In the logistic regression model, crude odds ratios (cORs) were calculated with their respective 95% CI, considering a *p*-value < 0.05 as statistically significant. Subsequently, adjusted odds ratios (aORs) were estimated to obtain the final regression model, with the aim of determining the risk and protective factors present in the analyzed sample.

## 3. Results

### 3.1. Sample Characteristics

A total of 425 women were included in the study, with a mean age at delivery of 32.65 years, a current mean age of 41.14 years, and a mean current BMI of 24.01 kg/m^2^.

Obstetric history showed that 77.41% of deliveries were vaginal (48.24% spontaneous and 29.18% instrumental), while 22.59% were cesarean sections. The episiotomy rate was 83.59%, and perineal tears were reported in 22.82% of cases. Overall, 25.88% of women reported pelvic floor dysfunction problems or symptoms related to UI, with a mean time to symptom onset of 2.8 years after childbirth.

After completing the validated ICIQ-UI-SF questionnaire in its Spanish-adapted version, 228 women (53.6%) were found to have UI, while 197 women (46.4%) did not. These two groups were used in the bivariate and multivariate analyses.

#### 3.1.1. Sociodemographic and Health Variables

Table 1 presents the sociodemographic and health characteristics of the participants according to the presence or absence of urinary incontinence.

Women with UI were older both at the time of delivery and at the time of evaluation. The mean age at delivery was 33.34 ± 4.85 years in the UI group and 31.86 ± 5.25 years in the non-UI group (*p* = 0.027), while the mean current age was 41.68 ± 5.00 years versus 40.51 ± 5.43 years (*p* = 0.0205). These findings indicate that older or more advanced maternal age, both at delivery and currently, is associated with a higher probability of developing IU years later. Most women were over 35 years of age at the time of the study, with a higher proportion in the UI group (89.47% vs. 81.22%).

No significant differences were found in educational level or occupation. Most participants had higher education, and approximately half had jobs that did not require physical effort.

The mean current BMI was slightly higher among women with UI (24.35 ± 4.44 vs. 23.62 ± 4.24), although the difference was not statistically significant (*p* = 0.086). Most women were in the normal weight to overweight range, whereas the prevalence of obesity was somewhat higher in the UI group (11.4% vs. 5.6%).

Family history of UI was significantly more frequent among affected women (29.52% vs. 16.75%; *p* = 0.002), suggesting a possible genetic or familial predisposition.

Likewise, women with UI showed a significantly higher frequency of personal medical history (36.4% vs. 26.4%; *p* = 0.027), which could contribute to worsen or exacerbate urinary incontinence.

No significant differences were observed in relation to physical activity habits, and the prevalence of constipation was similar in both groups (16.8% vs. 16.7%; *p* = 0.963).

Overall, maternal age, family history, and medical history showed a significant relationship with the presence of UI, whereas educational, occupational, and lifestyle variables showed no statistically significant associations.

#### 3.1.2. Obstetric and Perinatal Variables

Table 2 presents the results of the obstetric and perinatal variables. After analysis, no significant differences were found between the groups, except for the type of delivery.

A significant association was observed between the mode of delivery and the presence of UI. Vaginal delivery, both eutocic and instrumental, was more frequent among women with UI (81.14%) compared with those without UI (73.10%; *p* = 0.048). In contrast, the rate of cesarean section was higher in the non-UI group (26.90% vs. 18.86%). These findings suggest that vaginal delivery may play a relevant role in the subsequent development of urinary incontinence, probably due to its greater impact on pelvic floor musculature.

These results suggest a possible relationship between vaginal delivery and the subsequent development of UI, without significant influence from BMI, gestational weight, or episiotomy.

#### 3.1.3. Logistic Regression

Table 3 shows the crude and adjusted odds ratios with their corresponding 95% confidence intervals (95% CI) for the main risk and confounding factors associated with the occurrence of UI between 5 and 10 years after the first delivery.

In relation to maternal age at delivery, women who had their first childbirth after the age of 35 showed a significantly higher crude risk of developing urinary incontinence (UI) in the medium-to-long term (OR = 1.82; 95% CI: 1.07–3.09; *p* = 0.026). However, after adjusting for confounding factors, this association lost statistical significance (aOR = 1.20; 95% CI: 0.58–2.49; *p* = 0.629), suggesting that age alone does not constitute an independent risk factor once other variables are controlled for in the studied cohort. No significant associations were observed among women who had their first delivery between 30 and 35 years of age. Similarly, participants’ current age was not related to the presence of UI in either the crude or adjusted models.

Regarding current BMI, obese women (BMI > 30 kg/m^2^) showed a trend toward a higher risk of UI, although the association did not reach statistical significance (OR = 2.10; 95% CI: 0.64–6.87; *p* = 0.219; aOR = 2.25; 95% CI: 0.66–7.68; *p* = 0.195).

Mode of delivery showed a statistically significant association with the long-term development of UI. Women who had a cesarean section presented a lower risk of developing UI, both in the crude analysis (OR = 0.63; 95% CI: 0.40–0.99; *p* = 0.049) and in the adjusted model (aOR = 0.52; 95% CI: 0.32–0.85; *p* = 0.009). This result indicates a possible protective effect of cesarean delivery compared with vaginal birth.

The presence of a family history of urinary incontinence was significantly associated with a higher likelihood of developing UI between 5 and 10 years after the first childbirth (OR = 2.08; 95% CI: 1.30–3.33; *p* = 0.002; aOR = 2.03; 95% CI: 1.25–3.32; *p* = 0.004).

Similarly, the existence of relevant medical history—such as chronic diseases or comorbid conditions—was also related to an increased risk of UI (OR = 1.59; 95% CI: 1.05–2.42; *p* = 0.028; aOR = 1.58; 95% CI: 1.02–2.45; *p* = 0.040).

Overall, these results suggest that advanced maternal age at first delivery may initially be associated with an increased risk of medium- to long-term UI, although this effect appears to depend on concomitant factors. Conversely, cesarean delivery demonstrated a clearly protective role, while family and medical history remained independent and determining factors in the development of UI within 5 to 10 years after the first childbirth.

### 3.2. Urinary Incontinence Assessment

#### 3.2.1. ICIQ-UI-SF

Table 4 presents the final scores of the ICIQ-UI-SF questionnaire, which was completed by 100% of the study participants.

Among women with UI (N = 228), 149 exhibited moderates to severe incontinence, suggesting urine leakage of clinically relevant frequency and intensity.

According to the classification of UI types based on the ICIQ-UI-SF, 64.91% of the women presented symptoms consistent with SUI, making it the most frequent type. In contrast, 19.74% reported symptoms consistent with MUI, and 9.21% exhibited symptoms corresponding to pure UUI.

Finally, 6.14% of the women reported urine leakage related to other causes, not classifiable within the three main types described, such as: leakage during sleep, after voiding and dressing, or occurring unexpectedly or continuously. These results confirm the predominance of SUI in the studied cohort, with a relevant proportion of mixed forms suggesting the coexistence of combined pathophysiological mechanisms.

#### 3.2.2. Women’s Perception

**Figure 2:**
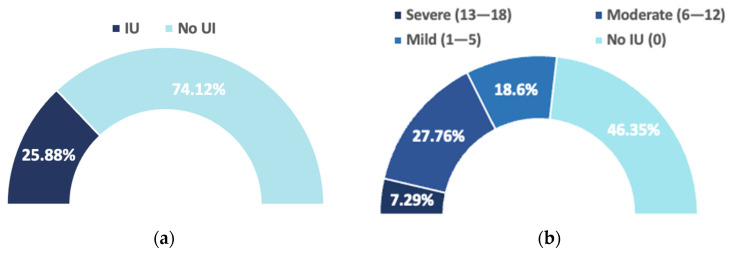
Perception of urinary incontinence. Open-ended question versus validated questionnaire: (**a**) Women perception; (**b**) ICIQ-IU-SF and distribution of urinary incontinence severity according to ICIQ-IU-SF.

In the initial interview, when participants were asked whether they had any pelvic floor problems or conditions related to urinary incontinence, only 25.88% answered affirmatively, reflecting a low self-perception of pelvic floor dysfunction (Figure 2a). However, after administration of the ICIQ-UI-SF questionnaire, the results showed that 53.65% of the women presented some degree of UI (Figure 2b). This finding highlights a significant discrepancy between subjective perception and the assessment obtained using a validated instrument.

Regarding the distribution of UI severity according to the ICIQ-UI-SF classification, 79 women presented mild incontinence, 118 moderate, and 31 severe, with no cases of very severe incontinence recorded (Figure 2b). Overall, moderate and severe categories accounted for 65.3% of all detected cases, underscoring the clinical relevance of the condition even several years after childbirth.

## 4. Discussion

We present the results on urinary incontinence as part of a broader study on pelvic floor dysfunctions conducted in our setting [36]. Overall, half of the women included in our study reported one or more PFDs within 10 years after childbirth. These figures are similar to those reported in the SWEPOP study [28] and the EPICONT study [20], although they differ from prevalence studies conducted in the United States [43]. However, these studies compare populations with different characteristics and use varying instruments to measure prevalence. Nevertheless, all concur that urinary incontinence is the most prevalent pelvic floor dysfunction.

From a methodological and validity standpoint, the use of validated instruments—particularly those that are culturally and linguistically adapted, such as the Spanish version of the ICIQ-UI-SF employed in our study—ensures that measurements are reliable and comparable across diverse populations, thereby enhancing the consistency and robustness of our estimates [41]. In addition, standardized data collection by specialized healthcare professionals (midwives) helped to minimize measurement variability. Nevertheless, the use of telephone interviews may have introduced recall or social desirability bias, and the observational design of the study inherently limits causal inference.

However, strength of our study lies in its focus on primiparous women with no subsequent deliveries, thus avoiding the confounding effect of later births—well-documented risk factors in the literature [43,44]—and restricting the analysis to women of reproductive age, acknowledging the known influence of aging on this condition [34,43,44].

In our cohort, just over half of the evaluated women (53.65%) presented some degree of UI according to the ICIQ-UI-SF, a higher prevalence than that reported in the Spanish meta-analysis (30–40%) [34].

Regarding factors associated with the onset of UI, and in line with most authors, we consider vaginal delivery the main risk factor for SUI. Thus in our series 3 out of 4 women with UI (74.12%) had as a triggering component of their incontinence the effort alone or accompanying the urgency. The cause is believed to be related to irreversible damage to the pelvic floor, including loss of pelvic organ support [45], levator ani muscle injury [46], and pudendal nerve dysfunction [47]. Consequently, cesarean delivery appears to play a protective perineal role.

This hypothesis is supported by observational studies reporting a higher prevalence of UI after vaginal delivery compared with cesarean section [17,20,28,48], as well as meta-analyses demonstrating an increased risk of postpartum UI following vaginal birth compared to cesarean delivery [49,50,51].

In our cohort, women with pre-pregnancy UI were excluded to limit the analysis to a single pregnancy and delivery, as the pathophysiology of UI likely differs between women with pre-existing incontinence and those who develop it postpartum [29]. Indeed, a stronger protective effect of cesarean section has been found among continent women during pregnancy (RR 2.57; 95% CI: 2.17–3.04) compared with those who already experienced UI during pregnancy (RR 1.56; 95% CI: 1.27–1.92).

With respect to educational level, previous studies have linked lower education to a higher risk of UI, showing stronger associations in American populations [44] than in European cohorts [52], consistent with our findings.

In our sample, the role of body weight, analyzed through BMI at pregnancy or at the time of the interview, did not emerge as a risk factor for UI. This may be due to the characteristics of our cohort (mean age at delivery: 32.65 years; mean age at interview: 41.14 years; mean BMI: 24.01 kg/m^2^), which differ from studies involving older and heavier populations [53,54], factors generally associated with pelvic floor dysfunctions.

Family history (mother or sister) appeared as an independent risk factor for UI in our cohort. This likely reflects a combination of genetic and environmental influences. In a case–control study conducted in an American population [55], the prevalence of SUI among mothers was 34.9% (71/203) in the study group compared to 12.7% (19/149) in the control group (*p* < 0.005). Among sisters, the prevalence of SUI was 19.9% (73/367) in the study group and 6.8% (15/220) in controls (*p* < 0.005). Similarly, in Europe, data from the EPINCONT study [56] showed that women were more likely to develop UI if their mothers (OR = 1.3; 95% CI: 1.2–1.4; absolute risk: 23.3%) or older sisters (OR = 1.3; 95% CI: 1.2–1.4; absolute risk: 23.3%) were incontinent.

These familial aggregation studies point toward a genetic basis for UI. Research has identified up to 11 genes implicated in the pathogenesis of SUI; however, genome-wide studies are still required to confirm these genetic associations. Such findings may lay the groundwork for future development of novel therapies and biomarkers for SUI in clinical practice [57].

In our study, medical history (29.52%, n = 67) also behaved as an independent risk factor—for example, through conditions such as diabetes or via medications like diuretics used to treat hypertension. In a cross-sectional population-based study conducted in Nord-Trøndelag County, Norway (1995–1997) [58], the prevalence of UI among women with diabetes was 39%, compared to 26% among non-diabetic women. Diabetic women exhibited higher frequencies of urge and mixed incontinence. Associations between diabetes and urge incontinence (OR = 1.49; 95% CI: 1.03–2.16), mixed incontinence (OR = 1.32; 95% CI: 1.05–1.67), and severe incontinence (OR = 1.54; 95% CI: 1.21–1.96) remained significant after adjusting for age, BMI, parity, and smoking.

On the other hand, the onset of transient urinary incontinence, detected during the interview secondary to well-known promoting factors such as diuretic use, have also been described in the literature, particularly among older adults [59].

In our study, a nutritional survey (e.g., beverage, coffee, or tea consumption) was not performed, as it fell outside the study’s primary objectives. Other known risk factors (physical activity, constipation, among others) were analyzed but did not reach statistical significance.

In the analyzed cohort, 53.65% of women exhibited some degree of UI according to the ICIQ-UI-SF. One quarter (27.76%) had moderate symptoms and 7.29% severe, with no cases classified as “very severe.” By UI type, SUI predominated, followed by MUI and UUI. These findings emphasize that a considerable proportion of women experience clinically relevant symptoms years after childbirth, particularly in moderate/severe cases (65.35% of those with UI). The distribution of types and severity burden is consistent with previous literature [60].

Regarding the temporal evolution of SUI after the first delivery, Arrue et al. (2021) documented that cumulative incidence increased from 14.2% at 6 months to 39.6% at 12 years, with predominantly mild–moderate severity and low impact on quality of life for most women [12]. These data help contextualize the predominance of moderate cases in our cohort and their likely clinical trajectory.

The impact on health-related quality of life correlates with severity. Both Spanish studies using the King’s Health Questionnaire (KHQ) and population-based reviews demonstrate that greater symptom severity is associated with worse quality of life and higher psychosocial burden. Although our study used the ICIQ-UI-SF as a measure of symptom severity and impact rather than the KHQ, the high proportion of moderate/severe scores suggests a clinically relevant impact in a subgroup of women—consistent with the findings of Martínez Córcoles et al. (2008) in Spanish healthcare settings [4]. Similarly, the PURE study (Prospective Urinary Incontinence Research)—a longitudinal, observational, multicenter study across 15 European countries—identified UI severity as the strongest predictor of impaired HRQoL and discomfort, regardless of UI type [61].

In this regard, Ros et al. (2015) [6], in a Spanish population study, reported that even when UI symptoms impair quality of life, many women neither seek consultation nor treatment. Only a small proportion of women with UI seek professional help, and this likelihood increases with symptom severity and coexistence of other PFDs. However, the perception that it is “a normal part of aging,” skepticism regarding treatment effectiveness, and the associated stigma act as barriers [6]. Moreover, healthcare professionals often fail to inquire proactively about continence status. Thus, this gap between need and demand is crucial to interpret prevalence estimates based on validated instruments such as the ICIQ-UI-SF.

Our findings, derived from a standardized and validated tool, are consistent with robust national and international evidence [12,60,62]. However, self-perception of UI is often underestimated due to normalization or stigma, suggesting that ICIQ-UI-SF scores likely reflect the true burden more accurately than non-validated questionnaires. This aligns with the Spanish study by Ros et al. [6], which reported that many women adapt to UI by using pads or modifying lifestyle habits rather than seeking care, emphasizing the value of active screening.

From a preventive perspective, particularly in this reproductive-age population—before aging introduces additional risk factors—several measures can be considered:

(a) Pelvic floor muscle training (PFMT) during early pregnancy has been shown to have positive effects on continence capacity postpartum [63].
(b) Cesarean delivery appears to have a protective effect on female pelvic floor dysfunctions, including UI. The Cochrane Review by Lavender et al. (2012) examined this issue as a long-term secondary maternal outcome and concluded that there is no evidence from randomized controlled trials to support elective cesarean section for non-medical reasons at term [64].
(c) Preventive measures, including weight control, dietary modifications (avoiding excessive fluid intake and consumption of caffeine or tea), and management of comorbid or contributing conditions (e.g., chronic diseases or related treatments), may help reduce UI risk. Lifestyle modifications and bladder training may also be beneficial for certain UI types [65].
(d) In general, universal screening for UI in asymptomatic women is not recommended due to insufficient evidence regarding its effectiveness and potential harms. However, opportunistic screening in primary care has been proposed in Spain [66] by scientific societies (SEMERGEN, SEMG, and semFYC) for asymptomatic women over 40 years of age. In line with our results, targeted screening may be useful in selected groups of women to facilitate preventive interventions.
(e) Finally, there is high certainty evidence that pelvic floor muscle training can cure symptoms and improve quality of life across all UI types [65].

## 5. Conclusions

In middle-aged, non-menopausal women without prior urinary incontinence, the onset of UI in the years following childbirth is associated with a history of vaginal delivery, maternal family antecedents, and the presence of chronic diseases or comorbidities. Therefore, given the high prevalence and significant impact on quality of life observed, we recommend implementing preventive measures with strong evidence before the onset of age-related biological changes, including menopause.

## Figures and Tables

**Table 1:** Characteristics sociodemographic and health variables of the cohort of women who do not have urinary incontinence (UI) and those who have UI.

	No UI ^1^ (mean/sd) (%) N = 197	UI (mean/sd) (%) N = 228	*p*
**Age at delivery**	31.86/5.25	33.34/4.85	0.027
<30	28.43%	21.05%	
30–35	47.21%	46.05%	0.081
>35	24.37%	32.89%	
**Age current**	40.51/5.43	41.68/5	0.0205
<30	3.05%	1.75%	
30–35	15.74%	8.77%	0.053
>35	81.22%	89.47%	
**Educational Level**			
High	81.22%	79.39%	
Low	18.78%	20.61%	0.636
**Job occupation**			
No physical effort	52.79%	54.39%	
Physical effort	46.70%	45.61%	0.539
**Current BMI ^2^**	23.62/4.24	24.35/4.44	0.0863
<18.5	4.06%	3.95%	
18.5–29.9	90.36%	84.65%	0.105
>30	5.58%	11.40%	
**Family history**			
No	83.25%	70.48%	
Yes	16.75%	29.52%	0.002
**Medical history**			
No	73.60%	63.60%	
Yes	26.40%	36.40%	0.027
**Physical exercise**			
No	26.40%	29.82%	
Yes-High impact	21.83%	18.86%	0.636
Yes-Low impact	51.78%	51.32%	
**Constipation**			
No	83.16%	83.33%	
Yes	16.84%	16.67%	0.963

^1^ UI, urinary incontinence; ^2^ BMI, body mass index.

**Table 2:** Obstetric and perinatal characteristics of the cohort of women who do not have urinary incontinence (UI) and those who have UI.

	No UI ^1^ (mean/sd) (%) N = 197	UI (mean/sd) (%) N = 228	*p*
**Pre-pregancy BMI ^2^**	22.66/3.72	23.01/3.89	0.35
<18.5	8.12%	5.26%	
18.5–29.9	86.80%	89.04%	0.485
>30	5.08%	5.70%	
**BMI at delivery**	27.26/4.21	27.71/4.28	0.2828
<18.5	0.51%	0.00%	
18.5–29.9	79.70%	73.68%	0.167
>30	18.80%	26.32%	
**Weight gain (kg)**	12.12/5.64	12.47/6.33	0.548
**Gestational age (weeks)**	39.21/1.83	38.98/2.10	0.2334
**Infant birthweight (g)**	3202/548	3163/585	0.4769
<3000	31.98%	32.89%	
3000–3999	61.42%	62.28%	0.73
>4000	6.60%	4.82%	
**Type of birth**			
Vaginal (instrumental + eutocic)	73.10%	81.14%	
Cesarean section	26.90%	18.86%	0.048
**Episiotomy (N = 330)**			
No	19.31%	14.59%	
Yes	80.69%	85.41%	0.254

^1^ UI, urinary incontinence; ^2^ BMI, body mass index.

**Table 3:** Crude (cOR) and adjusted Odds Ratios (aOR) and 95% Confidence Intervals (CI) of possible risk factors for urinary incontinence.

Risk/Confounding Factors	Crude OR (95% CI)	*p*	aOR (95% CI) ^1^	*p*
**Age at delivery**				
30–35	1.32 (0.81–2.12)	0.257	0.86 (0.43–1.72)	0.671
>35	1.82 (1.07–3.09)	0.026	1.20 (0.58–2.49)	0.629
**Current age**				
30–35	0.97 (0.24–3.86)	0.963	1.05 (0.25–4.34)	0.944
>35	1.91 (0.53–6.89)	0.322	2.31 (0.55–9.71)	0.254
**Current BMI ^2^**				
18.5–29.9	0.96 (0.37–2.55)	0.941	0.93 (0.34–2.55)	0.882
>30	2.10 (0.64–6.87)	0.219	2.25 (0.66–7.68)	0.195
**Type of birth**				
Cesarean section	0.63 (0.40–0.99)	0.049	0.52 (0.32–0.85)	0.009
**Family history**				
Yes	2.08 (1.30–3.33)	0.002	2.03 (1.25–3.32)	0.004
**Medical history**				
Yes	1.59 (1.05–2.42)	0.028	1.58 (1.02–2.45)	0.04

^1^ A total of 424 women contributed to the model; ^2^ BMI, body mass index.

**Table 4:** Assessment of urinary incontinence using the International Consultation on Incontinence Questionnaire–Urinary Incontinence Short Form (ICIQ-UI-SF). Final score.

ICIQ-IU-SF Final Score	N	%
No urinary incontinence (0)	197	46.35
Mild (1–5)	79	18.6
Moderate (6–12)	118	27.76
Severe (13–18)	31	7.29
Very severe (19–21)	0	0

## Data Availability

The data presented in this study are available on request from corresponding author.

## Abbreviations

The following abbreviations are used in this manuscript:

aOR	Adjusted Odds Ratio
BMI	Body Mass Index
ICD	International Classification of Diseases
ICIQ-UI-SF	International Consultation on Incontinence Questionnaire–Urinary Incontinence Short Form
ICS	International Continence Society
IUGA	International Urogynecological Association
KHQ	King’s Health Questionnaire
HRQoL	Health-Related Quality of Life
MUI	Mixed Urinary Incontinence
PFDs	Pelvic Floor Disorders
SEMERGEN	Spanish Society of Primary Care Physicians
SEMG	Spanish Society of General and Family Physicians
semFYC	Spanish Society of Family and Community Medicine
SUI	Stress Urinary Incontinence
UI	Urinary Incontinence
UUI	Urgency Urinary Incontinence
WHO	World Health Organization
